# Seroprevalence and Associated Factors of Human Immunodeficiency Virus, *Treponema pallidum*, Hepatitis B Virus, and Hepatitis C Virus among Female Sex Workers in Dessie City, Northeast Ethiopia

**DOI:** 10.1155/2021/6650333

**Published:** 2021-05-25

**Authors:** Yeshi Metaferia, Abdurahaman Ali, Solomon Eshetu, Daniel Gebretsadik

**Affiliations:** ^1^Department of Medical Laboratory Science, College of Medicine and Health Sciences, Wollo University, P.O. Box 1145, Dessie, Ethiopia; ^2^Beza Posterity Development Organization, Dessie, Ethiopia

## Abstract

**Introduction:**

Sexually transmitted infections (STIs) are prevalent in Ethiopia and elsewhere among different population groups particularly among female sex workers (FSWs). Because of their work and their behavior, FSWs are at high risk to acquire STIs. The aim of the study was to assess the seroprevalence and associated factors of HIV, HBV, HCV, and *T. pallidum* among FSWs in Dessie City, Northeast Ethiopia.

**Methods:**

This cross-sectional study was conducted in Dessie City, Amhara Region, Northeastern Ethiopia, from November 2017 to April 2018. A total of 360 FSWs whose age is greater than or equal to 18 years and who are willing to participate were recruited by simple random sampling technique. Interview-based questionnaire was administered, and 5 ml of venous blood from each participant was drawn under aseptic conditions. The rapid test was performed to obtain the result of the four STIs (HIV, *T. pallidum*, HBV, and HCV). The collected data were entered and analyzed by SPSS version 20.0. From the bivariable analysis, variables having *P* value < 0.2 were retained into multivariable analysis. From the multivariable analysis, variables with *P* value < 0.05 were affirmed as statistically associated factors. Adjusted odds ratios and their 95% confidence intervals were used as indicators of the strength of association.

**Results:**

Majority of study participants were urban dwellers, 10 (2.8%) respondents were married, 61 (16.9%) have more than two children, and more than half of them were at the age range between 18 and 27 years. Any infection with STIs was 84 (23.3%), whereas 27 (7.5%), 47 (13.1%), 2 (0.6%), and 45 (12.5%) study participants were positive for laboratory test of HIV, HBV, HCV, and *T. pallidum*, respectively. Marital status, sharing of sharp materials, breakage of condom, number of customers per week, genital discharge, and pain had significant association with any STI.

**Conclusions:**

In comparison with different research works in Ethiopia and abroad, the prevalence of any STI, HIV, HBV, and *T. pallidum* was found to be relatively high. Preventive approach and appropriate treatment of STIs should be developed. Concerned body should work together to alleviate the problem by counseling and recruiting them on other productive job sectors in the country.

## 1. Introduction

Sex workers include females and males who received money or goods in exchange for sexual service either regularly or occasionally [1]. Sex work may vary in the degree to which it is formal or organized. It is important to note that sex work is consensual sex between adults, which takes many forms and varies between and within countries and communities. Because of their work and their behavior, female sex workers (FSWs) are at high risk to acquire sexually transmitted infections (STIs). For example, they do not want to attend voluntary HIV counseling and testing service, and higher levels of alcohol consumption which is practiced by most FSWs have been associated with having never tested for HIV [2–5]. Utilization of alcohol and not knowing HIV status of women at higher sexual risk are the determinant factors for high prevalence of HIV [6].

Human immunodeficiency virus (HIV), hepatitis B virus (HBV), hepatitis C virus (HCV), and *T. pallidum* infections are considered as the cause of sexually transmitted diseases among males and females [7]. Their infections and other STIs like gonorrhea, human herpes virus (HHV), and human simplex virus (HSV) are the most important infectious diseases in developing and developed countries [8–14]. Although sexual contact is the prime mode of transmission, they are also transmitted through exposure to infected body fluids and from mother to fetus or child during the prenatal period [9, 15, 16]. Different risky behaviors and sexual behaviors make the community particularly the youth at the greatest possibility to acquire some of the most prominent STIs like HIV, HBV, and HCV [17].

Sexually transmitted infections are prevalent in Ethiopia and elsewhere among different population groups particularly among FSWs. Their prevalence among FSWs was associated with different factors such as having genital sore, age, marital status, educational level, duration as FSW, early sexual debut, and number of clients [15, 18, 19]. In sub-Saharan Africa, the estimated proportions of FSWs among women were 0.4% to 4.3% with the possibility of changing the figure through the course of time [20].

Based on the published articles in different parts of the globe, the prevalence of HIV among FSWs ranges from 0.9% to 73.7% [4, 18, 19]. The prevalence of HCV, HBV, and *T. pallidum* was also significant in such kind of population group [13–15]. Similarly, a study conducted among FSWs in Rwanda indicated 51.1%, 2.5%, and 1.4% overall prevalence for syphilis, HBV, and HCV, respectively [18]. According to the World Bank report, HIV prevalence among FSWs varied significantly by region, with the highest prevalence found in sub-Saharan Africa (36.9%) and followed by Eastern Europe (10.9%). It has been revealed that HIV prevalence among this group of population was 13.5 times higher than the overall HIV prevalence among the general population of women 15–49 years old [21]. In Nepal, the prevalence of HBV, HCV, and HBV-HCV coinfections among HIV-positive patients was found to be 3.6%, 2.9%, and 0.3%, respectively. This study revealed that a higher CD4+ T cell count had an association with a decreased risk of HCV or HBV infection [22].

HIV prevalence in the general population of a given country has strong association with the number of HIV-infected FSWs [23]. This is because they can transmit for their client, marital partner, or cohabitants and for their children. In addition, they have the habit to move from one prostitution area to another and FSWs who have practice in mobile prostitution sites had a higher rate of exposure for STIs [24]. Due to this and other scenarios, Ethiopia may face strong challenge in controlling HIV and other STIs. A declining trend in the overall prevalence of syphilis and HBV among pregnant women in Ethiopia from 2005 to 2014 was revealed [10]. In Ethiopia at large and in Amhara Region specifically, there was limited data on the seroprevalence of HBV, HIV, HCV, and *T. pallidum* among FSWs. In the study area, the extent of STIs and determinant factors are not clearly identified and documented particularly on the most at risk groups like FSWs. In order to fill the epidemiological data gap about STIs among FSWs, there is a need to conduct research that focuses on different aspects of the problem. The main aim of the current study was to assess the seroprevalence and associated factors of HIV, HBV, HCV, and *T. pallidum* among FSWs in Dessie City, Northeast Ethiopia.

## 2. Material and Methods

### 2.1. Study Design, Area, and Period

A cross-sectional study was conducted in Dessie City, Amhara Region, Northeastern Ethiopia, from November 2017 to April 2018. Dessie is the capital city of south Wollo zone and is located in the northeastern part of Ethiopia, at approximately 401 km from the capital city, Addis Ababa. The exact number of FSWs in the city is unknown due to the dramatic incremental opening of new bars, hotels, restaurants, coffee houses, etc. As a result, the number of females as sex workers is alarmingly increasing by considering it as a better job opportunity and a good source of income. However, the 2017 data from the employees and social affairs office of Dessie City revealed that the number of FSWs is around 2162.

### 2.2. Study Populations

FSWs registered in Dessie City employees and social affairs office who were visiting Beza Posterity Development Organization Dessie site were selected.

### 2.3. Sample Size Determination

A single population proportion formula was used to estimate the sample size, and the following assumption was considered: 95% confidence interval (*Z*_*α*/2_ = 1.96), 50% proportion was taken because there is limited data about STIs among FSWs in the area and nearby localities, and 5% margin of error.

(1) $$\begin{array}{c} n=\frac{{\left(Za/2\right)}^{2}P\left(1-P\right)}{d^{2}}=384=\text{ni}. \end{array}$$

But the total number of source population is less than 10000, so the above calculated number of study participants was processed with reduction (correction) formula nf = ni/(1 + (ni/*N*)) = 384/(1 + (384/2162)) = 326; by adding 10% nonresponse rate, the final sample size was 359~360 female sex workers.

### 2.4. Inclusion Criteria

Only female sex workers registered in Dessie City employees and social affairs office and who were visiting Beza Posterity Development Organization Dessie site were included in the study. We have considered and defined women as FSWs when they are living and commercializing sex for the last 6 months in Dessie City. Female sex workers with age greater than or equal to 18 years old and who are willing to participate in the present study were included.

### 2.5. Exclusion Criteria

Those female sex workers who were with apparent mental or physical illness that limit them from interview and sex workers who are not available during the study period were excluded.

### 2.6. Sampling Technique

Simple random sampling technique was used to select 360 study participants during the study period. Prior to the actual data collection, we have studied the average number of flow of study participants. Then, the number of study participant who were going to be included in the study in each day was allocated.

### 2.7. Data Collection Methods

After obtaining an informed and written consent, adopted and modified standardized questionnaires were used to collect the information from each study participant through interview method. An interview-based questionnaire was administered in order to collect the sociodemographic, behavioral, and other predisposing related variables that are associated with the dependent variable. 5 ml of venous blood was drawn under aseptic conditions by the data collectors. The tube was labeled and processed at the time of collection. The rapid test was performed to obtain the result of the four STIs (HIV, HBV, HCV, and *T. pallidum*). The data collection has taken place at Beza Posterity Development Organization Dessie site.

### 2.8. HBsAg, Anti-HCV, and Anti-*T. pallidum* Detection

A one-step rapid in vitro qualitative sandwich immunochromatographic diagnostic test kit was used for the detection of HBsAg, anti-HCV, and anti-*Treponema pallidum* in patient's serum. The test procedure and result interpretation were carried out according to the manufacturer's instructions.

### 2.9. HIV Serology

HIV screening was carried out using the rapid diagnostics test kits, by adopting national testing algorithm. A sample from each study participant was being first tested using Wantai HIV kit (Beijing Wantai Biological Pharmacy Enterprise Co., Ltd.). All positive samples were further tested using the Unigold HIV kit (Trinity Biotech Plc, Ireland), and positive results were presumptively considered as positive. The Unigold test kit is used as second line (confirmatory), and when it is negative, the Stat-Pak HIV kit (a tie breaker) (Chembio USA) was then used. All test kit procedures have been done according to their respective manufacturer's instruction.

### 2.10. Data Processing and Analysis

The collected data was entered into “EpiData 3.1” software and exported to SPSS version 20.0 (SPSS, Chicago, IL, USA) for analysis. Descriptive statistics like frequencies and proportions were used to summarize the data. Bivariable and multivariable analyses were used to examine the relationship between the dependent and independent variables (sociodemographic characteristics, behavioral factors, and predisposing factors). Variables which show significance at *P* value of 0.2 during bivariable analysis were selected for multivariable analysis. Adjusted odds ratios (AOR) and their 95% confidence intervals (CIs) were used as indicators of the strength of association. *P* value < 0.05 was used to indicate statistical significance.

## 3. Results

### 3.1. Sociodemographic-Related Information

A total of 360 FSWs participated, and out of these, only 8 (2.2%) had their education up to diploma and above level. Majority of them were urban dwellers, about 10 (2.8%) respondents were currently married, 61 (16.9%) have more than two children, and more than half of them were at the age range between 18 and 27 years (Table 1).

### 3.2. Risk Factor-Related Information

More than half, 212 (58.9%), of the study participants had awareness about the transmission and prevention mechanisms of STIs; majority of them had no surgical procedure history, history of blood transfusion, sharing of sharp materials, and substance abuse behavior. Almost all study participants had nose/ear piercing experience and alcohol drinking habit (Table 2).

### 3.3. Serological Test Results and Associated Factors

Among the total 360 FSWs, 27 (7.5%), 47 (13.1%), 2 (0.6%), and 45 (12.5%) were positive for HIV, HBV, HCV, and *T. pallidum*, respectively. Any infection with these STIs was 84 (23.3%) whereas coinfection of HIV, HBV, and *T. pallidum* was found on six study participants. The prevalence of both HIV and HBV infection was higher in the age group between 38 and 47 years (19.4%) (Table 3).

More than 20 variables were assessed for the presence of association with any STI. In the bivariable analysis, age, marital status, price per customer, place where customer was obtained, having children, occupation other than commercial sex work, awareness about STIs, hospital admission history, surgical operation history, history of blood transfusion, sharing of sharp materials, tattooing, genital mutilation, alcohol drinking habit, frequent drug abuse, age at which sexual contact started, history of condom breakage, number of customers in one week, presence of discharge, and duration of being FSWs had shown a *P* value < 0.2. But in the multivariable logistic regression model, marital status, sharing of sharp materials, breakage of condom, number of customers per week, genital discharge, and pain had a significant association with any STI at *P* value < 0.05 (Table 4).

Many different variables were assessed for the presence or absence of association with HIV, HBV, and *T. pallidum* infection using both bivariable and multivariable logistic regression models. The bivariable analysis of the three infections was computed independently, and we have used a cutoff *P* value of 0.2 to recruit and analyze the variables in the multivariable model. In the multivariable logistic regression model, marital status, sharing of sharp materials, presence of genital discharge, and pain during the data collection period had significant association with HIV infection. There was only one variable (history of breakage of condom) that had significant association with HBV infection. Age, marital status, having children, sharing of sharp material, history of breakage of condom, and genital discharge variables had significant association with *T. pallidum* at *P* value < 0.05 (Table 5).

For HIV-HBV coinfection, sharing of sharp materials (AOR = 6.48, 95%CI = 1.12–37.44, *P* = 0.037) and presence of genital discharge (AOR = 7.03, 95%CI = 1.25–39.66, *P* = 0.027) were factors that had significant association. Old age FSWs were more likely to be affected by HIV-*T. pallidum* coinfection; those FSWs who had a habit of sharing sharp material had more odds of acquiring the coinfections. The sole determinant factor for high HIV-HBV-*T. pallidum* coinfection was the presence of genital discharge during their visit to the health care facility (Table 6).

## 4. Discussion

In this study, the seroprevalence of HIV, HBV, HCV, and *T. pallidum* was determined among FSWs in Dessie City and its overall prevalence of at least one of the four STIs was found to be 84 (23.3%). This finding is higher than a study conducted in Brazil [25] among FSWs (13.3%) and among female prisoners in Indonesia (17.8%) [26]. Among the total study participants, 27 (7.5%), 47 (13.1%), 2 (0.6%), and 45 (12.5%) were positive for HIV, HBV, HCV, and *T. pallidum*, respectively. A study conducted in Rwanda indicated a higher prevalence of *T. pallidum* (51.1%), HIV (42.9%), and HCV (1.4%), but the prevalence of HBV (2.5%) was lower in comparison with the current study [18]. In a study conducted in Argentina, the seroprevalence of HIV, HBV, HCV, and *T. pallidum* was found to be 3.2%, 14.4%, 4.3%, and 45.7%, respectively [27].

Like a study conducted in Rwanda [18], older age was identified as an associated factor of high HIV and *T. pallidum* coinfection in the current study. The same factor was found to be a predictor for the prevalence of single infection with *T. pallidum* in the current study, which was in agreement with a study conducted in Argentina and China [27, 28]. Marital status of the study participants had a statistically significant association with the seroprevalence of at least any one of the four STIs, single infection of HIV and *T. pallidum*. Being single, divorced, and widowed had protective association for the seroprevalence of any STI. Although marital status of FSWs showed association in the bivariable analysis, it did not persist as a determinant factor for the HIV infection in a study conducted in the Central African Republic [29]. In Ethiopia, one study indicated that divorced marital status had significant association with high HIV infection [9]. Another study in Uganda indicated that being widowed is an independently associated factor for high prevalence of HIV among women who were involved in high-risk sexual behavior [6].

Another similar study in Italy showed the seroprevalence of the above STIs was 4.6% for HIV, 3.5% for HBV, 2% for *T. pallidum*, and 0.9% for HCV [13]. Similarly, a study conducted in the Republic of Congo showed a comparable proportion of HIV and HCV with the current study whereas the seroprevalence of HBV and *T. pallidum* was lower [24]. The seroprevalence of HIV-HBV coinfection (2.8%), HIV-*T. pallidum* coinfection (4.4%), and HIV-HBV-*T. pallidum* coinfection (1.7%) was indicated in the present study. The HIV-*T. pallidum* coinfection was higher in Rwanda [18]; on another way, a study conducted in Libya reported zero prevalence of HIV-HBV coinfection [30] and a comparable HIV-HBV coinfection (3.6%) was reported among HIV patients in Nepal [22].

In the current study, seroprevalence of HIV infection and *T. pallidum* among FSWs was 27/360 (7.5%) and 45 (12.5%), respectively. In similar studies that have been conducted in Brazil, Panama, Kenya, and Democratic Republic of Congo [15, 16, 31, 32], the prevalence of *T. pallidum* was lower than that of the current study. Another study in Brazil revealed the seroprevalence of HIV was almost in agreement with the present study whereas the prevalence of *T. pallidum* (19.7%) was high [11]. A much lower prevalence of both *T. pallidum* and HIV infection was also reported from similar studies in England and China [33, 34]. Even though it was conducted just before 20 years, a study in Ethiopia had reported a very high prevalence of HIV (73.7%) and *T. pallidum* (52.4%) [19]. This increased rate of HIV and *T. pallidum* might be because of the lack of awareness, inaccessibility of health care service for FSWs, and study population selection bias during the study.

In the current study, the presence of discharge during their visit at the health care facility is a strong positive predictor for any STI (AOR = 7.66), single infection for HIV (AOR = 10.2) and *T. pallidum*, and coinfection of HIV-HBV, HIV-*T. pallidum*, and HIV-HBV-*T. pallidum*. In a study conducted in Rwanda, recent history of genital sores was indicated as factor for high HIV-*T. pallidum* coinfection [18]. History of sharing sharp materials among FSWs had near to 3 times more odds of acquiring any STI. It had also association with single infection of HIV (AOR = 3.6) and *T. pallidum* (AOR = 12.7) and coinfection of HIV-HBV (AOR = 6.48) and HIV-*T. pallidum* (AOR = 8.4). In contrast to the present finding, a study done in Ethiopia reported this factor did not show association with seroprevalence of either HIV or HBV [9].

According to the result of a systematic review and ecological analysis among FSWs in Europe, the prevalence of HIV and *T. pallidum* was considerably lower than that in the current study [35]. In the contrary, a higher pooled prevalence (11.8%) of HIV infection was indicated in a systematic review and meta-analysis that was conducted in low- and middle-income countries [36]. Other studies in Mekelle city, Ethiopia (11.9%) [37], in Democratic Republic of Congo (12.4%) [16], in Kenya (16.4%) [15], and in Central Africa Republic (19.1%) [29] also indicated that higher proportions of FSWs were acquiring HIV infection. The difference of these research findings might be due to the variation in study period, source population, laboratory method, legal framework of the countries, and demographic features. In disagreement with the current study, history of condom breakage was documented as a significant factor for high HIV infection in Mekelle city, Ethiopia [37].

The seroprevalence of HBV and HCV in this research work was 47 (13.1%) and 2 (0.6%), respectively. A study conducted in Brazil revealed that their prevalence was 9.3% and 0.5%, respectively [38]. A study conducted in Ethiopia showed 6% of FSWs were infected with HBV [37]. In the contrary, a study that has been conducted in Libya indicated 0% and 5.2% seroprevalence for HBV and HCV, respectively [30]. In disagreement with the current study, a higher prevalence of both HBV (18.2%) and HCV (10.6%) was reported in a study conducted in Burkina Faso [39]. In another similar study that has been conducted in Nigeria, a higher rate of HBV was indicated [40]. This variation in seroprevalence of HBV and HCV among different research works might be due to the difference in the nature of population, study period, pattern of risky sexual behavior of the index cases such as condom reuse with clients, socioeconomic status, and cultural variations.

History of condom breakage during sexual intercourse with their client had 3.28 times chance to acquire any STI in comparison to their counterpart. It has also a positive significant association with independent infection of HBV (AOR = 3.45) and *T. pallidum* (AOR = 9.99). Unlike the current study, this factor did not show significant association with HBV infection in Mekelle city, Ethiopia [37]. According to a study on condom utilization and sexual behavior of FSWs in Ethiopia, having lower number of clients per month was positively associated with condom utilization, even though condom utilization practice by FSWs was low [41]. Good condom utilization is one of the important STI prevention mechanisms. However, in the present study, it has revealed that those having 1-3 customers per week were more likely to be infected with STIs than those FSWs who had greater than 3 customers per week (AOR = 2.05, 95%CI = 1.036–4.053, *P* = 0.039). It might be due to the possible occurrence of condom breakage among this group of FSWs; as we have explained above, history of condom breakage had a significant association with STIs. A study that has taken place in Burkina Faso did not report any association of the number of clients per week with HBV and HCV infection [39]. Having 1-2 children was identified as an associated factor for low rate of *T. pallidum* infection (AOR = 0.06, 95%CI = 0.009–0.403) in comparison with those FSWs who do not have children. But this factor did not demonstrate any association with other STIs including HIV, HBV, and HCV. Number of children did not show association with HIV infection in a study conducted in Central African Republic [29]. Similar studies had reported that having children did not demonstrate association with HBV infection [39] and HIV-*T. pallidum* coinfection [18].

A lower proportion of HIV (2.5%) and HBV (1.9%) infections was reported in a study that aimed at determining HIV and HBV infections in young women attending health institutions for abortion care [9]. In comparison with the current study, a lower seroprevalence of HIV (4.1%), *T. pallidum* (1.9%), and their coinfection (0.7%) was indicated among pregnant women in Gondar [42]. Another lower seroprevalence of HIV and HBV was indicated in studies that were conducted among blood donors in Ethiopia. These studies also showed a higher rate of HCV seroprevalence in comparison with the current study [43, 44]. In Amhara Region, Ethiopia, the overall incidence rate of HIV from 2015 to 2018 was 6.9 per 1000 population and the proportion of females who were infected with HIV was higher (59%) and the age group between 25 and 49 years was the major part of the population with the infection. This study also indicated that the incidence rate of HIV per 1000 population was high in Dessie City (5.7) which showed that the prevalence of HIV in the current study has increased about 13-fold than the report [45].

The seroprevalence of HIV, HBV, and *T. pallidum* in the current study was higher than that in another vulnerable group of the population in different parts of the globe. The seroprevalence of HIV, HBV, and *T. pallidum* among female inmates in Indonesia was 3.7%, 3.3%, and 7.0%, respectively [26], and a reduced seroprevalence of HIV and HBV among men having sex with men was also reported in Libya [30]. Similarly, a much lower proportion of HBV seroprevalence was indicated among health care workers and medical waste handlers in Ethiopia [46]. In another way, a higher seroprevalence of HIV, HBV, and HCV in comparison with the current study was reported among people who inject drug (PWID) in Mozambique [12]. The rate of HIV among PWID was found higher than their counterpart [35]. Since the means of transmission of HBV and HCV are similar with HIV, their prevalence, particularly among PWID, could be possibly high. Significantly higher rate of HIV and comparable proportion of *T. pallidum* among women who are involved in high-risk sexual behavior were indicated in a study conducted in Uganda [6].

## 5. Conclusion

A total of 360 commercial female sex workers participated in this study, and the seroprevalence of any one of the four STIs, HIV, HBV, HCV, and *T. pallidum*, and their coinfections was determined. In comparison with different research works in Ethiopia and abroad, the prevalence of any STI, HIV, HBV, and *T. pallidum* was found to be relatively high. Different factors like sociodemographic, behavioral, clinical, and previous history-related information have been also assessed for the presence of association with specific infections and coinfections of the four STIs. Sharing of sharp materials, breakage of condom, number of customers per week, having children, genital discharge, and pain were some of the predictor variables for a single or combination (coinfection) of the abovementioned infections. Since they can be a source of infection for the community, first, a mass screening activity on FSWs should be done. Then, a preventive approach and appropriate treatment of STI scheme should be developed. Finally, the government, any other nongovernmental organizations, civic society, and religious institutions should work together to alleviate the problem by counseling and recruiting them on other productive job sectors that are found in the country.

## 6. Limitation of the Study

In this study, there are few limitations that we come across. The study subjects included in this study were FSWs who were coming to the health facilities. Since we did not address FSWs who are not coming to the health facility, there might be the gap of representativeness. In fact, we have used the national testing algorithm, but due to budget restraint, a confirmatory test using ELISA, TPHA, and other methods was not performed. In addition, this research did not address the seroprevalence of STIs among male clients of FSWs.

## Figures and Tables

**Table 1:** Sociodemographic characteristics of female sex workers (*N* = 360) at Dessie City from November 2017 to April 2018.

S. no.	Parameters	Frequency	Percentage
1	Age in years		
	18-27	202	56.1
	28-37	122	33.9
	38-47	36	10
2	Educational background		
	Illiterate	96	26.7
	1-8	187	51.9
	9-12	69	19.2
	Diploma and above	8	2.2
3	Occupation		
	Sex worker with additional occupation	113	31.4
	Only sex worker	247	68.6
4	Birth place		
	Urban	293	81.4
	Rural	67	18.6
5	Marital status		
	Married	10	2.8
	Single	188	52.2
	Divorced	132	36.7
	Widowed	30	8.3
6	Do you have children		
	1-2	92	25.6
	>2	61	16.9
	No	207	57.5
7	Income per individual		
	1-500 birr	225	62.5
	>500 birr	135	37.5
8	Place they found customer		
	Home	68	18.9
	Night club	204	56.7
	Street	88	24.4
9	Duration of work as FSW		
	1 to 2 yrs	139	38.6
	>2 years	221	61.4
10	Age (in years) when being FSW started		
	≤20	179	49.7
	>20	181	50.3

**Table 2:** Risk factor-related characteristics of female commercial sex workers (*N* = 360) at Dessie City from November 2017 to April 2018.

S. no.	Parameters	Frequency	Percentage
1	Awareness about STIs		
	Yes	212	58.9
	No	148	41.1
2	History of hospital admission		
	Yes	113	31.4
	No	247	68.6
3	Ever had history of surgical procedure		
	Yes	42	11.7
	No	318	88.3
4	History of blood transfusion		
	Yes	31	8.6
	No	329	91.4
5	Sharing of sharp materials		
	Yes	54	15
	No	306	85
6	Presence of tattoo in a body part		
	Yes	147	40.8
	No	213	59.2
7	Ear/nose piercing		
	Yes	341	94.7
	No	19	5.3
8	Genital mutilation		
	Yes	152	42.2
	No	208	57.8
9	Drinking alcohol		
	Yes	323	89.7
	No	37	10.3
10	Drug abuse		
	Yes	34	9.4
	No	326	90.6
11	History of breakage of condom		
	Yes	268	74.4
	No	92	25.6
12	Number of customers per week		
	1-3	128	35.6
	>3	232	64.4
13	Presence of discharge		
	Yes	44	12.2
	No	316	87.8

**Table 3:** Age distribution of HIV, HBV, HCV, and *T. pallidum* and their coinfection among female sex workers at Dessie City from November 2017 to April 2018. Note: the percentage in each age category was computed within age categories.

STI categories	18-27 yrs		28-37 yrs.		38-47 yrs.		Total	
	No.	%	No.	%	No.	%	No.	%
HIV	6	3.0	14	11.5	7	19.4	27	7.5
HBV	25	12.4	15	12.3	7	19.4	47	13.1
HCV	1	0.5	1	0.8	0	0	2	0.6
*T. pallidum*	12	5.9	18	14.8	15	41.7	45	12.5
HIV+HBV	2	1	5	4.1	3	8.3	10	2.8
HIV+*T. pallidum*	2	1	7	5.7	7	19.4	16	4.4
HIV+HBV+*T. pallidum*	0	0	3	2.5	3	8.3	6	1.7
Any infection	35	17.3	32	26.2	17	47.2	84	23.3

**Table 4:** Bivariable and multivariable analysis of factors for any one of the four STIs among female sex workers (*N* = 360) at Dessie City from November 2017 to April 2018.

Parameter	Any STI		*P* value	COR [95% CI]	*P* value	AOR [95% CI]
	Positive No. (%)	Negative No. (%)				
Age			0.001		0.91	
18-27	35 (41.7)	167 (60.5)	Ref		Ref	
28-37	32 (38.1)	90 (32.6)	0.057	1.7 [0.99-2.9]	0.82	
38-49	17 (20.2)	19 (6.9)	0.000	4.3 [2.02-9.03]	0.66	
Marital status			0.000		0.048^∗^	
Married	8 (9.5)	2 (0.7)	Ref		Ref	Ref
Single	30 (35.7)	158 (57.2)	0.000	0.05 [0.01-0.24]	0.026^∗^	0.095 [0.012-0.76]
Divorced	34 (40.5)	98 (35.5)	0.003	0.09 [0.02-0.43]	0.006^∗^	0.05 [0.006-0.428]
Widowed	12 (14.3)	18 (6.5)	0.04	0.17 [0.03-0.92]	0.022^∗^	0.072 [0.008-0.682]
Price per customer						
1-500 birr	61 (72.6)	164 (59.4)	0.03	1.81 [1.06-3.1]	0.603	
>500 birr	23 (27.4)	112 (40.6)	Ref	Ref	Ref	
Place of obtaining customer			0.005		0.206	
Home	25 (29.8)	43 (15.6)	Ref	Ref	Ref	
Night club	36 (42.9)	168 (60.9)	0.16	1.64 [0.83-3.26]	0.707	
Street	23 (27.4)	65 (23.6)	0.099	0.61 [0.33-1.1]	0.112	
Having children			0.005		0.861	
1-2	26 (31)	66 (23.9)	0.034	1.87 [1.05-3.34]	0.998	
>2	22 (26.2)	39 (14.1)	0.002	2.68 [1.42-5.05]	0.646	
No	36 (42.9)	171 (62)	Ref	Ref	Ref	
Having another occupation						
Yes	40 (47.6)	73 (26.4)	0.000	2.53 [1.53-4.19]	0.12	
No	44 (52.4)	203 (73.6)	Ref	Ref	Ref	
Awareness about STIs						
Yes	55 (65.5)	157 (56.9)	0.16	1.44 [0.86-2.39]	0.918	
No	29 (34.5)	119 (43.1)	Ref	Ref	Ref	
Hospital admission history						
Yes	36 (42.9)	77 (27.9)	0.01	1.94 [1.17-3.22]	0.265	
No	48 (57.1)	199 (72.1)	Ref	Ref	Ref	
Surgical operation history						
Yes	19 (22.6)	23 (8.3)	0.001	3.22 [1.65-6.26]	0.889	
No	65 (77.4)	253 (91.7)	Ref	Ref	Ref	
Blood transfusion history						
Yes	16 (19)	15 (5.4)	0.000	4.09 [1.93-8.7]	0.301	
No	68 (81)	261 (94.6)	Ref	Ref	Ref	
Sharing of sharp materials						
Yes	30 (35.7)	24 (8.7)	0.000	5.83 [3.16-10.76]	0.009^∗^	2.96 [1.32-6.66]
No	54 (64.3)	252 (91.3)	Ref	Ref	Ref	Ref
Tattooing						
Yes	46 (54.8)	101 (36.6)	0.003	2.1 [1.28-3.44]	0.701	
No	38 (45.2)	175 (63.4)	Ref	Ref	Ref	
Mutilation						
Yes	43 (51.2)	109 (39.5)	0.058	1.61 [0.98-2.63]	0.907	
No	41 (48.8)	167 (60.5)	Ref	Ref	Ref	
Alcohol drinking habit						
Yes	79 (94)	244 (88.4)	0.143	2.07 [0.78-5.5]	0.357	
No	5 (6)	32 (11.6)	Ref		Ref	
Frequent drug abuse						
Yes	12 (14.3)	22 (8)	0.087	1.92 [0.91-4.08]	0.073	2.387 [0.921-6.182]
No	72 (85.7)	254 (92)	Ref	Ref	Ref	Ref
Age (in years) when sex started						
≤20	33 (39.3)	146 (52.9)	Ref	Ref	0.510	
>20	51 (60.7)	130 (47.1)	0.03	1.74 [1.06-2.86]	Ref	
History of breakage of condom						
Yes	76 (90.5)	192 (69.6)	0.000	4.16 [1.92-9]	0.012^∗^	3.28 [1.304-8.267]
No	8 (9.5)	84 (30.4)	Ref	Ref	Ref	Ref
No. of customers per week						
1-3	38 (45.2)	90 (32.6)	0.035	1.71 [1.04-2.81]	0.039^∗^	2.05 [1.036-4.053]
>3	46 (54.8)	186 (67.4)	Ref	Ref		Ref
Presence of discharge						
Yes	30 (35.7)	14 (5.1)	0.000	10.4 [5.17-20.91]	0.000^∗^	7.66 [3.094-18.989]
No	54 (64.3)	262 (94.9)	Ref	Ref	Ref	Ref
How long as FSW						
1-2 years	22 (26.2)	117 (42.4)	Ref	Ref	0.274	
>2 years	62 (73.8)	159 (57.6)	0.008	2.07 [1.21-3.57]	Ref	

Note: ^∗^statistically significant at *P* < 0.05; AOR: adjusted odds ratio; COR: crude odds ratio; Ref: reference category; 95% CI: 95% confidence interval.

**Table 5:** Associated factors for seroprevalence of HIV, HBV, and *T. pallidum* infection among female sex workers (*N* = 360) at Dessie City from November 2017 to April 2018.

Parameter	HIV		HBV		*T. pallidum*	
	*P* value	AOR [95% CI]	*P* value	AOR [95% CI]	*P* value	AOR [95% CI]
Age	0.358		NA		0.003	
18-27	Ref				Ref	
28-37	0.162				0.003^∗^	16.24 [2.54-103.7]
38-49	0.402				0.001^∗^	66.1 [5.84-747.4]
Educational status	NA		0.391		NA	
Illiterate			0.088			
1-8			0.097			
9-12			0.14			
Diploma and above			Ref			
Marital status	0.02^∗^		NA		0.07	
Married	Ref				Ref	
Single	0.322	0.32 [0.03-3.1]			0.021^∗^	0.044 [0.003-0.622]
Divorced	0.01^∗^	0.05 [0.005-0.482]			0.013^∗^	0.037 [0.003-0.501]
Widowed	0.012^∗^	0.04 [0.003-0.48]			0.12	0.103 [0.006-1.815]
Price per customer	NA		NA			
1-500 birr					0.353	
>500 birr					Ref	
Place of obtaining customer	0.774		NA		0.12	
Home	Ref				Ref	
Night club	0.507				0.217	
Street	0.910				0.471	
Having children	0.481		NA		0.009^∗^	
1-2	0.633				0.004^∗^	0.06 [0.009-0.403]
>2	0.255				0.463	0.546 [0.108-2.75]
No	Ref				Ref	
Having another occupation						
Yes	0.105		0.168		0.653	
No	Ref		Ref		Ref	
Awareness about STIs	NA					
Yes			0.784		0.344	
No			Ref		Ref	
Hospital admission history	NA					
Yes			0.862		0.255	
No			Ref		Ref	
Surgical operation history						
Yes	0.612		0.8		0.317	
No	Ref		Ref		Ref	
Blood transfusion history						
Yes	0.129		0.311		0.730	
No	Ref		Ref		Ref	
Sharing of sharp materials						
Yes	0.028^∗^	3.6 [1.15-11.34]	0.087	2.05 [0.901-4.673]	0.000^∗^	12.73 [3.36-48.15]
No	Ref		Ref		Ref	
Tattooing						
Yes	0.354		0.052	2.103 [0.994-4.451]	0.065	0.31 [0.09-1.077]
No	Ref		Ref		Ref	
Mutilation	NA					
Yes			0.871		0.072	0.33 [0.096-1.11]
No			Ref		Ref	
Ear piercing			NA		NA	
Yes	0.544					
No	Ref					
Alcohol drinking habit	NA				NA	
Yes			0.097	6.145 [0.72-52.45]		
No			Ref			
Frequent drug abuse	NA				NA	
Yes			0.101			
No			Ref			
Age when sexual contact started			NA			
≤20 years	Ref				Ref	
>20 years	0.327				0.286	
History of condom breakage						
Yes	0.388		0.039^∗^	3.45 [1.067-11.159]	0.038^∗^	9.99 [1.13-88.04]
No	Ref		Ref		Ref	
No. of customers per week	NA					
1-3			0.417		0.396	
>3			Ref		Ref	
Presence of discharge						
Yes	0.000^∗^	10.2 [2.82-37.123]	0.348		0.000^∗^	121.1 [22.96-639.06]
No	Ref		Ref		Ref	
How long as FSW			NA			
1-2 years	Ref				Ref	
>2 years	0.140				0.234	

Note: ^∗^statistically significant at *P* < 0.05; AOR: adjusted odds ratio; COR: crude odds ratio; Ref: reference category; 95% CI: 95% confidence interval; NA: not applicable to multivariable analysis due to the bivariable analysis *P* value > 0.2.

**Table 6:** Associated factors for HIV-HBV, HIV-*T. pallidum*, and HIV-HBV-*T. pallidum* infection among female sex workers (*N* = 360) at Dessie City from November 2017 to April 2018.

Parameter	HIV+HBV		HIV+*T. pallidum*		HIV+HBV+*T. pallidum*	
	*P* value	AOR [95% CI]	*P* value	AOR [95% CI]	*P* value	AOR [95% CI]
Age	0.757		0.06		0.639	
18-27	0.489	2.87 [0.145-56.52]	0.019^∗^	0.017 [0.001-0.507]	0.994	—
28-37	0.501	2.17 [0.227-20.75]	0.241	0.293 [0.038-2.28]	0.344	0.28 [0.02-3.92]
38-49	Ref		Ref		Ref	
Residence						
Urban	0.398	0.457 [0.07-2.8]	0.7	0.732 [0.149-3.59]	0.419	0.401 [0.044-3.678]
Rural	Ref		Ref		Ref	
Marital status	NA	NA	0.09		NA	NA
Married			0.047^∗^	64.8 [1.05-3992]		
Single			0.017^∗^	89.3 [2.27-3513.3]		
Divorced			0.284	3.27 [0.375-28.56]		
Widowed			Ref			
Price per customer	NA	NA			NA	NA
1-500 birr			0.434	0.381 [0.034-4.28]		
>500 birr			Ref			
Place of obtaining customer	0.78		0.382		0.46	
Home	0.531	1.95 [0.241-15.76]	0.813	1.265 [0.181-8.86]	0.268	5.15 [0.283-93.859]
Night club	0.568	2.15 [0.16-29.73]	0.218	0.221 [0.02-2.44]	0.857	1.372 [0.043-43.485]
Street	Ref		Ref		Ref	
Having children	0.453		0.431		NA	NA
1-2	0.271	4.78 [0.295-77.46]	0.987	1.02 [0.094-11.02]		
>2	0.63	2.09 [0.1-42.26]	0.367	3.35 [0.242-46.5]		
No	Ref		Ref			
Having another occupation						
Yes	0.342	3.43 [0.27-43.58]	0.407	2.31 [0.32-16.65]	0.984	0.968 [0.041-22.65]
No	Ref		Ref		Ref	
Awareness about STIs			NA	NA	NA	NA
Yes	0.211	4.8 [0.411-56.19]				
No	Ref					
Surgical operation history			NA	NA	NA	NA
Yes	0.247	0.112 [0.003-4.57]				
No	Ref					
Blood transfusion history			NA	NA	NA	NA
Yes	0.155	14.9 [0.358-620.7]				
No	Ref					
Sharing of sharp materials						
Yes	0.037^∗^	6.48 [1.12-37.44]	0.013^∗^	8.4 [1.56-45.15]	0.065	8.26 [0.88-77.57]
No	Ref		Ref		Ref	
Tattooing					NA	NA
Yes	0.702	1.476 [0.202-10.8]	0.872	1.15 [0.2-6.57]		
No	Ref		Ref			
Age when sexual contact started						
≤20 years	0.124	0.102 [0.006-1.87]	0.578	0.548 [0.066-4.54]	0.986	0.973 [0.046-20.65]
>20 years	Ref		Ref		Ref	
Presence of discharge						
Yes	0.027^∗^	7.03 [1.25-39.66]	0.000^∗^	52.39 [7.46-368.1]	0.02^∗^	15.5 [1.527-157.058]
No	Ref		Ref		Ref	

Note: ^∗^statistically significant at *P* < 0.05; AOR: adjusted odds ratio; COR: crude odds ratio; Ref: reference category; 95% CI: 95% confidence interval; NA: not applicable to multivariable analysis due to the bivariable analysis *P* value > 0.2.

## Data Availability

There are no material outputs from this study, and all data are those presented in the manuscript. The authors confirm that all data underlying the findings are fully available without restriction upon formal communication.

## Abbreviations

FSWs: Female sex workers
HIV: Human immunodeficiency virus
HBV: Hepatitis B virus
HCV: Hepatitis C virus
STIs: Sexually transmitted infections
PWID: People who inject drug.
